# Our Medicine

**Published:** 1856-05

**Authors:** 


### OUR MEDICINE.

THE medicine which we, the Editor of *Hall's New-York Journal of Health*, most unhesitatingly take for all the aches and ills that *our* flesh is heir to, which is uniformly successful, and which we almost daily recommend with the highest confidence, and which comes next in our esteem to the two great remedies of Air and Exercise, and which nobody but a Doctor can be induced to take, except now and then a sensible man among a million, is the TINCTURE OF TIME. The reason of its want of proper confidence is, that it costs nothing and has no mystery about it. What a grand thing it is for the Doctors that so few people have any sense, not even sense enough to take pains to keep well when they are so, *to keep well by doing justly, living temperately, and pursuing in moderation the various callings of human life.*

